# Uncovering the impacts of alternative splicing on the proteome with current omics techniques

**DOI:** 10.1002/wrna.1707

**Published:** 2022-01-03

**Authors:** Marina Reixachs‐Solé, Eduardo Eyras

**Affiliations:** ^1^ The John Curtin School of Medical Research Australian National University Canberra Australian Capital Territory Australia; ^2^ EMBL Australia Partner Laboratory Network and the Australian National University Canberra Australian Capital Territory Australia; ^3^ Catalan Institution for Research and Advanced Studies Barcelona Spain; ^4^ Hospital del Mar Medical Research Institute (IMIM) Barcelona Spain

**Keywords:** alternative splicing, proteome, RNA processing, transcriptomics, translatomics

## Abstract

The high‐throughput sequencing of cellular RNAs has underscored a broad effect of isoform diversification through alternative splicing on the transcriptome. Moreover, the differential production of transcript isoforms from gene loci has been recognized as a critical mechanism in cell differentiation, organismal development, and disease. Yet, the extent of the impact of alternative splicing on protein production and cellular function remains a matter of debate. Multiple experimental and computational approaches have been developed in recent years to address this question. These studies have unveiled how molecular changes at different steps in the RNA processing pathway can lead to differences in protein production and have functional effects. New and emerging experimental technologies open exciting new opportunities to develop new methods to fully establish the connection between messenger RNA expression and protein production and to further investigate how RNA variation impacts the proteome and cell function.

This article is categorized under:

RNA Processing > Splicing Regulation/Alternative Splicing
Translation > Regulation
RNA Evolution and Genomics > Computational Analyses of RNA

## Graphical abstract

Alternative splicing potentially leads to impacts on protein production and function. Multiple experimental and computational approaches have been proposed to measure how alternative splicing reshapes the proteome.

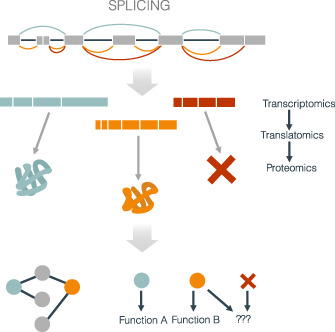

## 1 INTRODUCTION

With the human genome being almost complete, the estimated number of protein‐coding genes has stabilized at around 20,000 (Lander et al., 2001; Pertea et al., 2018). However, there is nearly six times the number of annotated proteins (115,885 in Ensembl GRCh38.p13, September 2021). The key to this diversity lies in the fact that every step in the production and processing of messenger RNA (mRNA), from transcription to translation, provides an opportunity for molecular diversification. Among all mRNA processing steps, perhaps the most well‐studied mechanism and possibly the one that provides the greatest chance for molecular variation in eukaryotes is splicing. First described in 1977, splicing is a sequential set of biochemical reactions to remove introns from the pre‐mRNA molecule (Berget et al., 1977; Chow et al., 1977). The discovery of splicing came with the observation that the same adenoviral gene produced different transcripts (Berget et al., 1977; Chow et al., 1977). The modular structure of the pre‐mRNA, with exons and introns, facilitates the assembly of different configurations of exons to generate alternative transcript isoforms (Gilbert, 1978), that is, alternative splicing. Soon after the discovery of splicing, the first case of alternative splicing was described in humans in the immunoglobulin μ gene, where one of the isoforms encoded the membrane‐bound antibody. In contrast, the other isoform encoded the secreted form of the protein (Singer et al., 1980). In recent decades, high‐throughput sequencing techniques have provided evidence indicating that more than 95% of the genes may undergo alternative splicing (Barbosa‐Morais et al., 2012; Pan et al., 2008; Wang et al., 2008). These results have highlighted the vast impact that alternative splicing has on shaping the transcriptome. However, it remains unclear how frequently transcriptome variation results in measurable protein and functional changes.

A shift in the relative abundances of the transcript isoforms produced from a gene locus may lead to the production of protein variants, which may in turn impact gene function through various mechanisms, including the alteration of protein–protein interactions (Ellis et al., 2012; Weatheritt et al., 2012; Wojtowicz et al., 2007). However, in vivo studies using cutting‐edge proteomics have failed so far to validate these functional impacts, sparking an intense debate on the field (Blencowe, 2017; Tress et al., 2017). In addition to the intrinsic differences and limitations of RNA sequencing and proteomics technologies, multiple regulatory mechanisms controlling transcript stability and translation could hinder the identification of direct links between transcript and protein variation (Braun & Young, 2014). Despite the abundant evidence supporting the functional impact of alternative splicing (Baralle & Giudice, 2017), whether it leads to fundamentally distinct proteins remains unknown for most genes. Understanding the regulation and interdependency of splicing with other regulatory processes is crucial to explain how the transcriptome encodes the functional complexity of cells. Moreover, to fill in the knowledge gaps, from gene expression to the production of different functional proteins, it is essential to gain insight into the mechanisms of RNA processing in the context of both the transcriptome and the translatome. In this review, we describe the mechanisms by which changes in RNA processing may result in the translation of different protein products, the high‐throughput techniques that have been developed to uncover these mechanisms, and the results from recent studies regarding the impact of alternative splicing in protein production and gene function.

## 2 MECHANISMS OF TRANSCRIPT DIVERSITY

**FIGURE 1:**
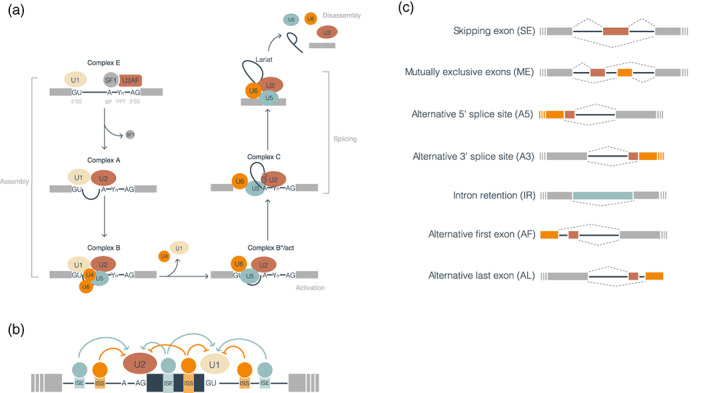
The mechanisms of splicing and alternative splicing. (a) The complexes and processing steps of splicing for the major spliceosome are depicted. In the assembly phase, U1, U2, U4, U5, and U6 are sequentially assembled in the mRNA molecule resulting in the formation of complex E, complex A, and finally, complex B. The activation phase consists of conformational and compositional rearrangements of complex B involving U2, U5, and U6. Two transesterifications occur as the catalytic complex C adapts its conformation: the first between the 5′ SS and the BP and the second between the 5′ end of the first exon and the 3′ end of the second exon. Finally, the snRNPs still attached to the mRNA disassemble to start a new cycle. (b) Illustration of the regulation of splicing by *cis*‐ and *trans*‐acting factors. Cis‐acting factors are represented as boxes in introns and exons. *Trans‐*acting factors are represented as circles bound to their corresponding *cis‐*acting elements: exonic splicing enhancer (ESE), exonic splicing silencer (ESS), intronic splicing enhancer (ISE), or intronic splicing silencer (ISS). Silencers are depicted in orange and enhancers in light blue. (c) Schematic representation of alternative splicing events. Gray boxes represent constitutive exons, red and orange boxes represent alternative exons, and a light blue box represents an exonized intron. Discontinuous ends in the exons indicate that the transcript continues in that direction. Thick blue lines represent introns in the transcript before processing, and dashed lines above and below represent the alternative processing of the exons

The splicing reaction starts with the recognition of sequence elements in the precursor messenger RNA (pre‐mRNA) by a small nuclear ribonucleoprotein (snRNP) complex, the spliceosome, which acquires its catalytic activity while assembling on the pre‐mRNA (Zhan et al., 2018; Figure 1a). In this process, intron sequences are excised from the pre‐mRNA, and exon sequences are spliced together to form the mRNA. Several sequence elements in the pre‐mRNA are essential for splicing. Most introns are characterized by the nucleotides GU at the 5′ splice site (SS) and AG at the 3′ SS, with very few exceptions (Figure 1a; Thanaraj & Clark, 2001). The branch point (BP) consists of an A nucleotide with a catalytic role near the 3’SS, and a polypyrimidine tract (PPT) is located between the BP and the 3′ SS. However, BPs may also occur far upstream of the 3′SS (Corvelo et al., 2010). The BP and PPT show specific sequence motif biases, but these may vary between species (Schwartz et al., 2008). The core spliceosome comprises five main snRNPs (U1, U2, U4, U5, and U6) that are sequentially assembled in the mRNA molecule, where U1 recognizes the 5′ SS and U2 the BP. Several conformational and compositional rearrangements involving U2, U5, and U6 take place to allow two transesterifications: the first one between the 5′ SS and the BP and the second between the 5′ end of the first exon and the 3′ end of the second exon (Figure 1a).

### 2.1 Alternative splicing regulation

Exon recognition is not straightforward, especially in metazoans, considering that the sequence surrounding the splice sites and the branch point can be highly variable, and introns are on average thousands of times larger than exons (Iwata & Gotoh, 2011; Lander et al., 2001; Long & Deutsch, 1999). For this reason, for efficient splicing to occur, additional *cis*‐acting elements on the pre‐mRNA sequence and *trans*‐acting proteins are often required (Figure 1b; Lim & Burge, 2001). These additional regulatory elements, which work together with the core splicing signals (3′SS, 5′SS, and BP), are not just essential for catalysis but often act as modulators of the splicing process. They are classified according to their location on intron or exons and their role in either enhancing or repressing splicing, resulting in four main classes: exonic splicing enhancer (ESE) or silencer (ESS), and intronic splicing enhancer (ISE) or silencer (ISS; Wang & Burge, 2008). Whereas most of these regulatory elements are located near the splice sites, distant ones have also been described to modulate exon inclusion (Lovci et al., 2013). Generally, these sequence motifs are short and represent binding sites for RNA binding proteins (RBPs; Fu & Ares, 2014). These RBPs are the *trans‐*acting factors that regulate exon definition and recognize the cis‐acting elements with variable affinity and specificity.

Two prominent RBP families that regulate splicing are SR proteins and heterogeneous nuclear ribonucleoproteins (hnRNPs), which often display opposite regulatory roles. SR proteins have one or two RNA‐recognition motifs and arginine/serine (RS)‐rich domains that mediate protein–protein interactions (Jeong, 2017). They generally bind ESEs to enhance exon inclusion but can exceptionally act as inhibitors by binding ISSs close to the intron boundaries (Bradley et al., 2015; Shen & Mattox, 2012). On the other hand, hnRNPs also show a modular structure with multiple RNA binding and auxiliary domains (Geuens et al., 2016). They bind ESSs to repress splicing, but as SR proteins, they can have an enhancing role depending on their position relative to other sequence elements, for example, by binding ISEs (Erkelenz et al., 2013). Multiple other RBPs have been found to play a role in splicing regulation in different physiological and disease contexts (Yee et al., 2019).

Numerous efforts have attempted to provide a complete census of proteins involved in splicing regulation, uncover their cognate sequence motifs, and establish their role in splicing, which have been jointly dubbed as the splicing code (Barash et al., 2010). These have been primarily based on cross‐linking and immunoprecipitation (CLIP) followed by high‐throughput sequencing, and have allowed identifying many RBPs and their recognition motifs, as well as other splicing regulators including coactivators or proteins involved in other RNA‐processing steps (König et al., 2011; Ule et al., 2003; Van Nostrand et al., 2020). However, the splicing code remains challenging to decipher due to the many proteins and sequence elements contributing to the splicing outcome. First, the strength of the splice sites, measured as their similarity to the consensus, dictates the susceptibility to regulation by *trans*‐acting factors. Strong splice‐site motifs facilitate splice‐site recognition, but weaker splice sites are more favorable for effective splicing modulation (Baralle & Baralle, 2018). Second, it is still challenging to determine if the binding of an RBP will positively or negatively regulate splicing since many of them have dual roles depending on the context (Fu & Ares, 2014). Finally, and most importantly, splicing patterns are driven by complex combinations of proteins and sequence motifs with cooperative and competitive relationships (Papasaikas et al., 2015; Ule & Blencowe, 2019).

### 2.2 Variation of the splicing products

The variation in splice site selection is generally described as local variations, which can be categorized into five primary events (Figure 1c; Box 1). The selection of alternative splice sites can make exons shorter or longer, generating alternative 5′ SS (A5) and alternative 3′SS (A3) splicing events. Splicing variation can also affect complete exons that may be spliced in or out of the mature transcript, described as a skipping exon (SE) event. Mutually exclusive exons (ME) are a complex case where one of two adjacent exons may be skipped, and they both are rarely observed together in a mature transcript. Finally, intron retention (IR) occurs when an intron is included in the mature transcript. Different combinations of primary events increase the number of isoforms produced from a single gene changing the UTRs or the coding sequence. Exons are classified as alternatives when they are differentially included in isoforms from the same gene, possibly described according to the event types mentioned above, or constitutive when present in all the transcript variants. On the other hand, these alternative splicing events coexist with other forms of transcript variation. The choice of alternative promoters can induce the selection of alternative first (AF) exons, and alternative polyadenylation (APA) can lead to the inclusion of alternative last (AL) exons (Figure 1c). Due to the involvement of regulatory RBPs that are common to splicing regulation, AF and AL events are often included when studying alternative splicing.

BOX 1 **Computational approaches for alternative splicing detection**

Genome‐wide detection of alternative splicing (AS) can be based on full‐length isoforms, exons, exon–exon junctions, or events. Isoform‐based methods use transcript abundances to calculate differential transcript usage (DTU), as in Cuffdiff2 from the Cufflinks package (Trapnell et al., 2010) or SUPPA (Alamancos et al., 2015; Trincado et al., 2018), or isoform switches, as in iso‐kTSP (Sebestyén et al., 2015). Both exon‐ and junction‐based analysis methods are generally based on short‐read counts. Exon‐based methods such as DEXSeq (Anders et al., 2012) identify differential usage of individual exons and exon fragments, which can mask splicing changes since they do not consider neighboring exons. Junction‐based methods, such as LeafCutter (Li et al., 2018), use short reads that span two consecutive exons. Event‐based methods like rMATS (Shen et al., 2014), MAJIQ (Vaquero‐Garcia et al., 2016), or SUPPA (Trincado et al., 2018) analyze alternative splicing choices represented in the form of events, as described in Figure 1c. Isoform and event‐based methods are easier to interpret in relation to the AS‐related functional impacts than methods based on exons or junctions. The inclusion level of an alternatively spliced event is measured as the Percentage (or proportion) Spliced In (PSI or Ψ), and its calculation varies depending on the method. For transcript isoforms, a relative abundance measure similar to PSI can be defined to study DTU and isoform switches (Climente‐González et al., 2017). Event‐ and isoform‐based approaches are complementary. However, splicing events are easy to interpret and validate experimentally, but they do not capture as much complex variation as using full‐length isoforms (Sebestyén et al., 2015). For this reason, in some cases, both analyses are implemented in the same tool. MISO provides a differential expression of events and isoforms from aligned RNA‐seq reads, whereas SUPPA (Alamancos et al., 2015; Trincado et al., 2018) uses transcript abundances for both isoform usage and PSI calculation, providing a direct correspondence between events and isoforms.

One or more alternative splicing events can give rise to a wide range of transcript‐isoform outputs, from two to a few hundred per gene (Ray et al., 2020; Tung et al., 2020), leading to complex patterns of coordinate splicing events (Grosso et al., 2008; Licatalosi & Darnell, 2010; Tapial et al., 2017). How they exactly contribute to functional diversification is still an active area of research. Complex alternative splicing patterns have been described to contribute to the definition of embryonic stem cells, to cell lineage differentiation processes, or to the epithelial–mesenchymal transition (Agosto & Lynch, 2018; Kalsotra & Cooper, 2011; Venables et al., 2013). Differentiated cells and tissues also show specific splicing patterns, as it has been described for the brain, heart, skeletal muscle, testis, and liver, as well as for erythrocytes and immune cells, among others (Baralle & Giudice, 2017; Bhate et al., 2015; Licatalosi & Darnell, 2010; Lynch, 2004; Nakka et al., 2018; Pimentel et al., 2016; Tapial et al., 2017). Finally, alternative splicing is also related to physiologic response to changing conditions, such as DNA damage response, thermal regulation, or even adaptation to stress conditions (Biran et al., 2020; Haltenhof et al., 2020; Shkreta & Chabot, 2015).

### 2.3 Interplay of splicing with other RNA processing steps

**FIGURE 2:**
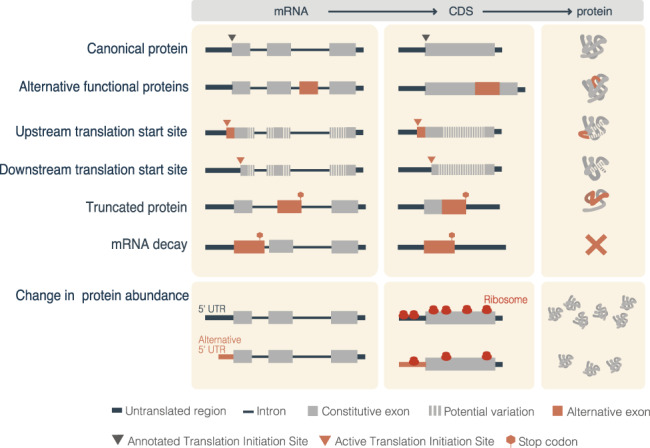
How alternative splicing potentially reshapes the protein products. Alternative splicing (AS) can reshape the proteome in diverse ways. These can be separated into two impacts: changing the protein sequence (upper panels) or altering the amount of protein produced (lower panels). Splicing changes affecting the coding sequences (CDS) can generate alternative proteins with different amino acid sequences, structures, and functions. Similarly, changes in the 5′ end of the mRNA molecule can lead to an alternative initiation site, which results in proteins with different amino‐terminal ends. Both mechanisms could also lead to a shift in the reading frame. Premature stop codons generated through a splicing change can translate into truncated proteins or lead to mRNA decay (Box 2). Finally, translation efficiency can be affected by the inclusion of alternative 5′UTRs through alternative splicing

During transcription initiation, expression of different transcription factors (TFs) and changes in chromatin state can lead to the selection of alternative promoters, thereby triggering transcription from an alternative transcription start site (Davuluri et al., 2008). As a result, different exons may be included at the 5′ end of the transcript that can modify the N termini of the protein (Leppek et al., 2018). This may happen as a direct sequence change of the open reading frame (ORF) of the resulting transcript (Tasic et al., 2002) or through a change in the translation start site mediated by alterations in the regulatory sequences of the 5′ untranslated region (5′UTR) of the mRNA (Figure 2). On the other hand, there is often no observable effect in the protein product. Instead, changes in the 5′UTR may affect the translation efficiency, that is, two or more of the transcript isoforms produced from the gene locus encode the same protein but at a different rate. (Tamarkin‐Ben‐Harush et al., 2017; Figure 2).

The coupling of splicing with transcription has been widely studied through the RNA Polymerase II (RNAPII) function. From the kinetic point of view, it has been shown that elongation rates of RNAPII can affect SS recognition with different outcomes depending on the SS strength (Fong et al., 2014; Saldi et al., 2017). On the other hand, the recruitment model is supported by known interactions of several SFs with RNAPII, mainly through the C‐terminal domain (CTD) of the large subunit or with other members of the elongation complex to regulate exon inclusion (Huang et al., 2012; de la Mata & Kornblihtt, 2006; Hsin & Manley, 2012; Das et al., 2007). For example, direct binding of U2AF on the CTD has been shown to recruit Prp19, crucial for spliceosome activation (David et al., 2011). A new approach in a recent study proposes multiple interactions between U1 and RNAPII that support a model of co‐transcriptional assembly of the spliceosome on the RNAPII (Zhang, Aibara, et al., 2021). Beyond RNAPII, it has also been observed that the use of alternative promoters, enhancers, and TFs affect splicing rates (Han et al., 2017; Alasoo et al., 2019).

Chromatin state has also been described as affecting the splicing outcome (Agirre et al., 2015, 2021; González‐Vallinas et al., 2015). Indeed, DNA methylation and histone posttranslational modifications (PTMs) can also modulate splicing and potentially participate in alternative splicing. Alternatively, spliced exons show lower methylation levels than constitutive ones (Gelfman et al., 2013). Moreover, depending on the factors mediating the crosstalk between methylation and splicing, the effect can be either promote or repress exon inclusion (Shukla et al., 2011; Maunakea et al., 2013; Yearim et al., 2015; Brito et al., 2020). Similarly, distinct histone modification signatures are also known to correlate with specific splicing patterns. This has been observed for *FGFR2*, where combinations of histone methylation marks regulate two mutually exclusive isoforms (Luco et al., 2010). This and other similar cases have led to the proposal of a chromatin code that can predict alternative splicing (Agirre et al., 2015, 2021).

Alternative polyadenylation (APA) is emerging as a relevant mechanism to regulate gene expression in multiple biological processes (Ren et al., 2020). APA can result in significantly different 3′ UTRs, affecting the transcript stability, export, and translation rates (Mayr, 2016; Kishor et al., 2020). Longer 3′ UTRs generally contain more regulatory structures and are related to decreased mRNA stability and translation efficiency (Moqtaderi et al., 2018). Moreover, if APA occurs upstream of the last exon, it can affect the 3′ UTR and the coding region by changing the exon composition at the 3′ end of the transcript. APA in coding regions can regulate gene expression and contribute to protein diversification (Tian & Manley, 2016). Apart from the core 3′ end processing factors, APA can be controlled by the interplay with other RNA‐processing steps, including transcription (chromatin state, TFs, and RNAPII activity) and splicing (Elkon et al., 2013; Gruber & Zavolan, 2019). RNA methylation dynamics have also been shown to regulate APA usage, promoting inclusion of terminal exons and therefore controlling the 3′ UTR length (Bartosovic et al., 2017).

Splicing regulators also influence other RNA biology processes that can alter the transcriptome beyond alternative splicing itself. For instance, members of the SR and hnRNP protein families can regulate transcription, processing, stability, and transport (Huang & Steitz, 2005; Ji et al., 2013; Geuens et al., 2016).

Furthermore, translation can be regulated to modulate the amount of protein produced and generate alternative proteins by the alternative selection of Translation Initiation Sites (TISs). Alternative TISs, often downstream of the canonical one, can be selected through several mechanisms, including leaky scanning of the ribosome, presence of non‐AUG codons, or through uORFs affecting the capacity of the ribosome to reinitiate translation (Kozak, 2005; Kochetov, 2008). In other cases, the start site of an uORF becomes the actual start site of a 5′ extended version of the translated ORF (Kochetov, 2008). In addition to the variation in the N‐termini of the protein, alternative TISs can also shift the translating frame, potentially resulting in different protein products and often creating premature stop codons (Orr et al., 2020).

Finally, biochemical RNA modifications are emerging as a mechanism to modulate the stability and translation efficiency of mRNAs (Boo & Kim, 2020). RNA modifications are abundant in ribosomal (rRNA), and transfer RNAs (tRNA), and their alterations impact translation through the functional impairment of rRNAs and tRNAs (Liu, Clark, et al., 2016; Ma, Wang, et al., 2019). There is now evidence that RNA modifications are also abundant in mRNAs (Dominissini et al., 2012; Squires et al., 2012), and the modifications 5‐methylcytidine and N4‐acetylcytidine have been linked to enhanced translation efficiency (Arango et al., 2018; Schumann et al., 2020; Huang et al., 2019). Given that there are more than 100 possible modifications (Linder & Jaffrey, 2019), this opens up multiple new possible mechanisms to modulate the protein output from mRNAs.

## 3 TOWARD IDENTIFYING THE EFFECTS OF SPLICING ON PROTEIN DIVERSITY

Transcriptomics studies have provided insight into how AS gives rise to transcript diversity in the cells. However, given the plethora of mechanisms to control and regulate protein synthesis after pre‐mRNA splicing (Box 2), the transcriptome is not a trustworthy representation of the proteome. Then, the key to determining the functional impact of AS is to understand how splicing events reshape the protein products but also to discriminate which alternative transcript isoforms will be translated into protein.

BOX 2 **Regulation of RNA processing after pre‐mRNA splicing**

Cellular mRNAs are subject to multiple layers of regulatory control. A significant proportion of this control occurs at the level of translation. Translation control is a primary regulator of gene expression that can act globally or specifically target certain mRNAs (Schwanhäusser et al., 2011). Global regulation occurs at the initiation step, where reversible protein phosphorylation controls the activity of initiation factors such as eIF2 and eIF4F (Jackson et al., 2010). Molecule‐specific regulation requires elements in the mRNA sequence able to influence translation, such as inhibitory secondary structures (hairpins), upstream ORFs (uORFs), or internal ribosome entry sequences (IRES; Gebauer et al., 2012). Other gene regulatory mechanisms involve small noncoding RNAs. Regulation by micro‐RNAs (miRNAs) is the most widespread one (Filipowicz et al., 2008), with thousands predicted to operate on about 60% of the protein‐coding gene in humans. Their predominant role is silencing by affecting the translation and stability of mRNAs (Friedman et al., 2009; Seiler et al., 2018). In addition to these regulatory mechanisms, there are quality control mechanisms to prevent the production of aberrant proteins that could accumulate and be detrimental to the cell. One of the most studied of these surveillance pathways is Non‐Sense Mediated Decay (NMD), which degrades mRNA molecules carrying a premature termination codon (PTC; Kervestin & Jacobson, 2012). Besides its function as a broad surveillance mechanism, there is evidence that NMD act as a specific gene expression regulator for functional transcripts (Nickless et al., 2017). Other ribosome‐associated surveillance pathways are Non‐stop decay (NSD), which degrades mRNAs lacking a stop codon (Frischmeyer et al., 2002), and No‐go decay, which targets mRNAs bound by stalled translation elongation complexes (Pisareva et al., 2011).

Most genes have one transcript that encodes the most ubiquitously expressed and evolutionary conserved protein isoform, and best represents that gene's function. This has motivated the definition of principal transcripts, which simplifies the transcriptome based on protein sequence, structure, function, and conservation (Rodriguez et al., 2013, 2018). However, this definition clashes with evidence indicating that the most expressed transcript isoform from a gene may vary between tissues and disease conditions (Gonzàlez‐Porta et al., 2013; Sebestyén et al., 2015). Changes in the most expressed protein‐coding transcript isoform can thus result in a diversification of proteins and their function, leading to changes in catalytic activities, interactions with other proteins, subcellular localization, and loss of function (Yang et al., 2016; Figure 2).

### 3.1 Proteomics evidence of alternative splicing

One of the primary sources of evidence to support the production of multiple proteins from the same gene locus through AS is mass spectrometry (MS; Blakeley et al., 2010). In a general MS workflow, proteins are digested with enzymes, and the generated peptides are ionized and loaded into a mass spectrometer. The generated spectra are used to computationally reconstruct peptide sequences using a database of annotated proteins as guide (Aebersold & Mann, 2003; Deutsch et al., 2008). Even though the existence of alternatively spliced isoforms from RNA‐seq analysis is incontestable, MS experiments have offered very little support for alternative protein products associated with AS, raising controversy in the field (Tress et al., 2017; Blencowe, 2017).

Although the detection power may vary significantly depending on the annotation database used, several studies have provided evidence for hundreds to about one thousand detectable alternative protein isoforms, often showing a tissue‐specific pattern (Wilhelm et al., 2014; Kim et al., 2014; Tay et al., 2015). A study of 30 human samples identified proteins corresponding to 84% of the total annotated protein‐coding genes and mapped peptides uniquely to approximately 400 alternative protein isoforms (Kim et al., 2014). In contrast, analysis of eight large‐scale human proteomics datasets with highly conservative filters yielded evidence for about 200 alternative protein isoforms (Ezkurdia et al., 2015; Abascal et al., 2015). It has also been claimed that there is an overestimation of alternative protein isoforms resulting from considering cases where peptides only map to one side of the variable exon in a splicing event rather than both sides, and from not taking into account the potential false positives in the MS data processing (Tress et al., 2017).

Although a significant amount of high‐throughput MS data have been produced to date for multiple cell models and tissues, studies using this data to search for protein isoform variants have almost invariably been performed in steady‐state conditions (Alfaro et al., 2017; Nusinow et al., 2020). This contrasts with the fact that most RNA splicing changes have been reported in association with changing physiological conditions or between normal and disease states. Supporting this notion, several studies have provided evidence for several genes with detectable protein isoform variation across the cell cycle in leukemia cells (Ly et al., 2014).

Integration of RNA‐seq experiments with MS for the identification of protein isoforms, also known as proteogenomics, has been implemented to reduce peptide mapping uncertainty and to detect novel peptides (Jeong et al., 2018; Liu et al., 2017; Wu et al., 2019; Lau et al., 2019; Agosto et al., 2019). In the context of depletion of a spliceosome component and using quantitative proteomics, it was shown that out of 450 transcripts with significant changes in RNA relative abundance, up to 160 showed consistent changes in protein production using quantitative proteomics (Liu et al., 2017). Similarly, a splice‐junction‐centric approach applied to public proteogenomics data led to the identification of 1500 alternative protein isoforms (Lau et al., 2019). Most of the junctions (~60%) arose from SE events, consistent with what has been observed in RNA‐seq experiments.

Proteogenomics studies have raised the issue of the poor correlation between transcript and protein abundances, which is usually below 0.5 (Pearson correlation coefficient) and varies across tissues (Kosti et al., 2016; Eraslan et al., 2019; Liu et al., 2017). Despite the general lack of consistency between transcript and protein abundances, it has been observed that above certain levels of transcript expression, RNA‐seq is a good predictor of protein expression (Vogel & Marcotte, 2012). A recent study has modeled the effect of protein synthesis regulation after pre‐mRNA splicing and has managed to explain 30% of the variance in the protein‐to‐mRNA ratios (Eraslan et al., 2019).

Many of the studies mentioned above point out that spectra generated in MS experiments might not provide enough resolution to distinguish particular isoforms (Tay et al., 2015; Liu et al., 2017; Kosti et al., 2016; Eraslan et al., 2019). On the one hand, these limitations could be attributed to the digestion enzymes used in proteomics. Trypsin is the standard protease in shotgun proteomics. It efficiently digests at K or R residues, resulting in small size (<3 kDa) peptides with a positive charge at the C‐terminus, which is optimal for the MS protocols (Huang et al., 2005; Laskay et al., 2013). The main drawbacks of the peptides produced are the short lengths (around six aa) and the limited proportion of the covered proteome (Giansanti et al., 2016). Other proteases such as chymotrypsin, LysC, LysN, AspN, GluC, ArgC, elastase, and proteinase K have been shown to cover complementary fractions of the proteome, and proteases such as GluC improve the detection of peptides containing PTMs, which are otherwise overlooked (Janssen et al., 2019); suggesting that combinations of enzymes may provide the best approach to obtain a comprehensive peptide identification (Dau et al., 2020; Giansanti et al., 2016). Regarding splicing products, different digestion enzymes used in proteomics are biased toward identifying certain splicing junctions with small overlap among them (Wang et al., 2018). On the other hand, since transcript isoforms share a considerable proportion of their sequence, isoform‐specific peptides are hard to identify (Tran et al., 2017). Nonetheless, proteogenomic approaches have improved the detection of peptides that can be uniquely matched to protein isoforms (Agosto et al., 2019). An added complexity in MS analysis is sequence identification from peptide mass spectra. Since the application of de novo peptide sequencing to MS remains challenging, the current analysis relies on database searches and can lead to false positives and neglect certain peptides (Bogdanow et al., 2016; Karunratanakul et al., 2019). In particular, a significant proportion of missed and unassigned peptides carry PTMs that shift the mass, which strongly impacts the database searches (Chick et al., 2015). Taken together, all these factors support the notion that there is an underestimation by MS experiments of the number of protein isoforms occurring in the cell.

### 3.2 Predicting the functional impact of alternative splicing from the sequence

In parallel to the proteomics approaches, other studies have tried to address the functional impact of alternative splicing using computational models to predict the function of the resulting coding and protein sequences. The most straightforward approach has been to analyze the effect of splicing events on the coding sequence (CDS). For instance, in VastDB (Tapial et al., 2017), predictions are classified into ORF‐preserving events, which have the potential to generate a fully functional protein, NMD targets, or ORF‐disrupting events, including both frame‐shifted and truncated CDS. However, these predictions are based on the impact of specific local events in isolation, without considering what happens elsewhere in the transcript, which does not reflect the complete splicing change. Indeed, a study of the functional impacts of isoform switches in cancer indicated that about 70% of these changes correspond to a single local splicing event. In contrast, the rest include changes that cannot be described in terms of the primary events (Climente‐González et al., 2017). Addressing this issue, the method ISOTOPE calculates the modified ORFs resulting from all the splicing alterations in the gene to calculate possible novel epitopes in tumor samples (Trincado et al., 2021).

Other studies have measured the impact in terms of the alteration in functional domains. Such approaches map protein domains and functional elements to transcript isoforms to identify which functions are lost or gained due to AS (Hiller et al., 2005; Sulakhe et al., 2019; de la Fuente et al., 2020; Climente‐González et al., 2017). For instance, one of these studies found that a large proportion of the isoform switches observed in cancer impacted functional domain families that are also targeted by cancer driver mutations (Climente‐González et al., 2017). In a different study, integrating the functional annotations with differential transcript usage (DTU) showed that ~80% of the genes with DTU between two different mouse neural precursor cells present differences in the content of the functional features (de la Fuente et al., 2020).

The impact on protein structure can also be used to evaluate the potential functional variability resulting from AS. Variable exonic regions have been observed to generally code for disordered regions in proteins, which has been interpreted as enabling functional and regulatory diversity as disorder regions confer conformational flexibility that can be accommodated in the context of structured proteins (Hegyi et al., 2011; Romero et al., 2006). Modeling the impact of splicing on protein structure faces significant challenges since very few protein isoforms have a resolved 3D protein structure, and it remains difficult to obtain structures for proteins without a stable fold (Melamud & Moult, 2009). To address these challenges, Hao et al. (2015) implemented a semi‐supervised learning approach based on structural information, and that does not require a negative set to conclude that 32% of alternatively protein isoforms result from SE events lead to functional proteins. Cross‐species conservation has also been used as evidence for the functionality of protein isoform variants (Mazin et al., 2021; Rodriguez et al., 2013; Tapial et al., 2017). In fact, phylogenetically conserved isoform variants generally show higher expression from mRNA and a higher detection rate in proteomics experiments than nonconserved ones (Ezkurdia et al., 2012; Cummings et al., 2020).

### 3.3 Effects on functional networks of protein interactions

One of the most intriguing functional effects of AS, and with potentially the farthest‐reaching consequences, is the modulation of protein–protein interaction (PPI) networks. It has been proposed that proteins with associated tissue‐specific splicing events are central hubs in interaction networks, having distinct interaction partners in different tissues (Buljan et al., 2012). Similarly, isoform switches in various cancer types have also been proposed to remodel PPIs involving cancer drivers and cancer‐related pathways (Climente‐González et al., 2017). Using in vitro experiments, it was observed that the inclusion of neuronally regulated exons significantly promoted or disrupted protein interactions using co‐immunoprecipitation assays (Ellis et al., 2012). In addition, yeast‐two‐hybrid experiments revealed how to splice variants expand the network of interactions in the autism spectrum disorders (Corominas et al., 2014). This mechanism was further studied at genome‐scale to reveal that less than 50% of interactions are shared among pairs of protein isoforms (Yang et al., 2016). Despite the vast range of the predicted isoform‐specific interactions in tissues and disease conditions, most PPI databases only capture the interactome of canonical protein isoforms and describe these in terms of one protein per gene. Recently, the database DIGGER has provided a way to retrieve interactions between domains and proteins at the isoform and exon levels (Louadi et al., 2021). Understanding the rewiring of functional networks driven by AS can shed light on how even minor changes in the mRNA and protein sequences can trigger significant functional effects on the cell.

All the described studies have added multiple layers of evidence for an impact of the transcriptome variation on the proteome and its functional repertoire that appears to be more extensive than what can be directly observed from proteomics experiments alone. However, one could argue that even controlling for NMD or using additional structural and functional information, most studies have been based on computational predictions or in vitro experiments and require further experimental validation using cell or in vivo models, circling back to proteomics and its limitations. Below we describe alternative methodologies that address these limitations.

## 4 TRANSLATOMICS: FILLING THE GAP BETWEEN MRNA AND THE PROTEIN

The basis for translatome analysis was already set more than 50 years ago (Warner et al., 1963; Steitz, 1969). However, it has been only recently that, thanks to combining those principles with high‐throughput sequencing, translatomics has emerged as a field with the potential to capture the elements involved in mRNA translation in a transcriptome‐wide manner (Zhao et al., 2019). We will focus on how translatomics techniques provide evidence of active translation of mRNAs and how this has been used to measure how AS can impact the proteome.

### 4.1 High‐throughput measurement of translating mRNAs

**FIGURE 3:**
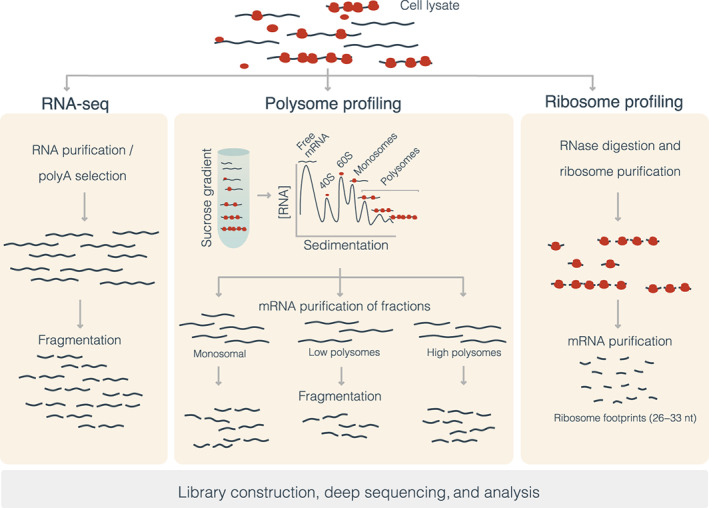
High‐throughput approaches to capture the translatome. RNA sequencing can capture all mRNAs in the cell lysate regardless of its translation status (left panel). Polysome profiling (middle panel) separates fractions of mRNAs according to the number of ribosomes attached to the mRNAs, which is assumed to correlate with the translation activity. Downstream sequencing of RNA in each polysomal fraction is performed as a standard RNA‐seq experiment, and the relative mRNAs abundances are interpreted as mRNA translation activities. In ribosome profiling or Ribo‐seq (right panel), mRNAs bound by the ribosomes are digested, producing footprints of approximately 30 nt representing the mRNA sites occupied by the ribosome. These fragments are then sequenced after detaching the ribosomes

The most widely used techniques for capturing translating mRNAs, polysome, and ribosome profiling, are based on the high‐throughput sequencing of transcripts bound by ribosomes (Figure 3). Polysome profiling experiments are based on sucrose gradient separation and ultracentrifugation of full‐length mRNAs, which have different sedimentation speeds according to the number of ribosomes sitting on them (Lackner et al., 2007; Mašek et al., 2011). This makes possible the stratification of different fractions containing mRNAs engaged by one (monosomes) or multiple (polysomes) ribosomes, indicating potentially different translation activities (Chassé et al., 2016; Floor & Doudna, 2016). Ribosomes are detached to perform high‐throughput sequencing in each fraction, like standard RNA sequencing for transcriptomics analyses, providing a straightforward measure of translation rates (Maslon et al., 2014). Ribosome profiling or Ribo‐seq follows a different approach (Ingolia et al., 2009). The segments of mRNA not bound by fully assembled ribosomes are digested, leaving intact only ribosome‐protected mRNA fragments of about 26–33 nucleotides (ribosome footprints), which are isolated for sequencing.

One advantage of Ribo‐seq over polysome profiling is that it provides the exact position where ribosomes are sitting in the mRNA molecule, that is, the ribosome footprint. Positional information enables the identification of start codon positions, codon composition effects on translation, detection of translating uORFs, or stalling (Zhang, Wang, et al., 2021; Ishimura et al., 2014). Moreover, the ribosome footprints can be assessed in terms of their uniformity along with the transcript and their deviations from the expected three‐nucleotide periodicity in the translation frames. Both measures are considered to be essential to determine active translation (Ji, 2018). However, Ribo‐seq experiments only capture fully assembled elongating ribosomes, that is, they do not provide information about the translation initiation and termination steps. A modification of the technique named translation complex profile sequencing (TCP‐seq) enabled to additionally retrieval footprints of the small ribosome subunit, expanding the picture to all stages of translation: initiation, elongation, and termination (Archer et al., 2016; Shirokikh et al., 2017). Translatomics approaches have been shown to significantly improve correlation with proteomics compared with transcriptomics‐based approaches (Wang et al., 2013; Blevins et al., 2019). Hence they represent promising alternatives to overcome the limitations of proteomics in the study of splicing‐associated alterations of the proteome.

### 4.2 Measuring alternative splicing from the translatome

Most of the initial studies on translatome profiling were conducted at gene level (Gonzalez et al., 2014; Ruiz‐Orera et al., 2014; Ingolia et al., 2009). However, as these experiments involve sequencing of transcriptome or transcriptome fragments, it is possible to apply transcript‐isoform‐aware tools to translatomics data to investigate how splicing impacts translation dynamics. In this regard, polysome profiling indicated that ~30% of splicing events show differences in polyribosome association that could be explained by remodeling of cis‐acting elements (Sterne‐Weiler et al., 2013). Similarly, translation regulation by SRSF1 was observed from polysomal fractions in mRNAs that display SRSF1‐dependent splicing changes, providing evidence for SRSF1 involvement in the coupling of nuclear mRNA processing events with translation (Maslon et al., 2014). Furthermore, different isoforms from the same gene have been shown to have variable translation potential during neural development using mRNAs from high polysome fractions (Blair et al., 2017).

There have also been several translatome studies seeking evidence of translation of alternative isoforms and AS events using Ribo‐seq experiments. In 2016, Weatheritt et al. mapped ribosome footprints across the exon–exon junctions of alternative splicing events, showing that 75% of skipping exon events from sufficiently expressed transcripts were engaged by the ribosomes (Weatheritt et al., 2016). However, it can be argued that ribosome engagement alone does not necessarily imply active translation of the full‐length isoform. To address this question, several groups have developed methods for isoform‐level quantification from Ribo‐seq data (Wang et al., 2016; Reixachs‐Solé et al., 2020; Calviello et al., 2020). ORFquant detected alternative ORFs being translated in some genes, although most genes showed one dominant isoform (Calviello et al., 2020). ORQAS pipeline showed higher sensitivity in detecting alternative isoforms, finding 33–53% of the expressed genes in mouse and human samples have more than one translated isoform. Additionally, 40% of the splicing changes at the RNA level were shown to be concordant with differences in translation (Reixachs‐Solé et al., 2020).

These and other studies have consistently reported that ribosome engaged splicing events are enriched in characteristics and functional elements that are relevant to specific physiological conditions, such as phosphorylation sites in regenerating liver, methylation islands in models of fragile X syndrome, or microexons (exons of length 3–51 nt) in glioma samples (Shah et al., 2020; Bhate et al., 2015; Reixachs‐Solé et al., 2020). These results also provided additional evidence for the contribution of alternative splicing to the definition of a specific cell, organ, and organismal phenotypes; and highlighted the relevance of studying the functional impact of alternative splicing in multiple physiological conditions and diseases.

Long read sequencing technologies enable the discovery and reconstruction of full‐length transcripts (Kovaka et al., 2019; Tang et al., 2020; Hu et al., 2021). This significantly improves the ability to identify potentially functional transcripts (Troskie et al., 2021) and provides the opportunity to define more accurate and complete references for translatomics studies (Wang et al., 2019; Sun et al., 2021). A recent study showed increased detection of actively translated alternative isoforms in rat hippocampus using full‐length transcripts compared to the available reference annotation (Wang et al., 2019). In another study, single‐molecule long‐read sequencing combined with ribosome profiles identified evidence of translation in RNAs in sperm that would be otherwise challenging to assemble using short sequencing reads (Sun et al., 2021). As long reads may solve the mapping uncertainty present in short reads, they could also improve ribosome profiling quantification by adapting existing methods that use transcript abundances as a guide to resolving ambiguity in Ribo‐seq mapping (Wang et al., 2016; Reixachs‐Solé et al., 2020).

## 5 CONCLUSION

Given the lack of accurate models to establish protein abundances from mRNA sequencing and the intrinsic limitations of the different approaches (summarized in Table 1), integration of multiple omics techniques addressing different stages of protein production remains to date the best strategy to study the impact of AS on the proteome and function. Although there is still some disagreement on the precise extent of the functional effects of AS, there is plenty of evidence that AS influences protein products. One of the most challenging obstacles is that existing proteomics methods may not provide enough sampling depth to keep up with the large number of splice variants identified from RNA‐seq. Therefore, it remains challenging to validate all the predicted variants at the protein level. An integral approach to overcome this limitation is to combine RNA‐seq information with proteomics analysis, which has been shown to significantly improve peptide matches (Ma, Liu, et al., 2019).

**TABLE 1:** Capabilities and limitations of current omics techniques for the detection of splicing variants

	Transcriptome	Translatome		Proteome
	RNA‐seq	Polysome profiling	Ribo‐seq	MS
Sample processing	RNase digestion or chemical fragmentation		RNase digestion	Protease digestion
Fragment length	75–150 nt		~30 nt	~10 aa
Sampling biases	Differences in PCR efficiency, overrepresentation of highly expressed mRNAs, lack of data for mRNAs with low expression.			Variable coverage depending on the protease used
Molecular biases	Underrepresentation of non‐poly A+ RNAs (in RNA‐seq with poly‐A+ selection), nuclear RNAs, lncRNA^a^			Underrepresentation of transmembrane proteins, PTMs, nonannotated variants, and translated noncoding regions
	Overrepresentation of rRNA (if rRNA depletion is not conducted), globin RNAs in blood samples, and so forth			Tissue‐specific overrepresentation of proteins
Splicing events detected	Events in coding regions, UTRs, and NMD targets	Events in coding regions, UTRs, and NMD targets^b^	Events in coding regions and NMD targets^c^	Events involving coding regions

^a^ lncRNA generally has low expression and are underrepresented but can still be translated into short peptides (Ruiz‐Orera et al., 2014).

^b^ NMD targets may still be engaged by the ribosome but not translated; hence they will be pulled down in monosome or low polysomal fractions (Heyer & Moore, 2016; Karousis et al., 2020).

^c^ With Ribo‐seq, elongating ribosomes can be discriminated from stalled ones using measures of periodicity and uniformity (Ji, 2018).

Measuring the relation between transcriptome and translatome changes has emerged as a powerful way to identify alternative splicing events that affect the protein product and, therefore, potentially impact function (Weatheritt et al., 2016; Reixachs‐Solé et al., 2020). Knowledge of the mRNA abundances from the transcriptome can help overcome the drawbacks of translatomics analysis. We can find a clear example of this in ribosome profiling. The short length of the fragments represents one of the main limitations for isoform quantification that can be addressed by considering isoform abundances from RNA‐seq (Wang et al., 2016; Reixachs‐Solé et al., 2020). At the same time, it has been shown that the incorporation of ribosome footprints as priors can improve the accuracy of protein isoform abundances estimated from MS (Carlyle et al., 2018). New protocols such as TCP‐seq have the potential to shed light on how splicing variation in noncoding regions (5′ UTR) can influence translation (Archer et al., 2016; Shirokikh et al., 2017). Thus, analysis of TCP‐seq data in an isoform‐aware fashion could uncover new mechanisms of translational control coupled with transcript initiation and alternative splicing. One potential limitation of polysome profiling and ribosome foot‐printing experiments is that they involve lengthy and complex protocols. Improvements on these protocols to make them easier to implement and combine with high‐throughput platforms could benefit the study of the impacts of alternative splicing on the proteome (Yoshikawa et al., 2021).

The integration of polysome profiling and ribosome footprinting with long‐read sequencing would be especially beneficial to the study of the translatome. Long‐read sequencing approaches would remove the uncertainty of transcript reconstruction and abundance estimation inherent in short reads. Full‐length transcripts and their abundances could be thus defined for the same conditions as in the Ribo‐seq experiments to describe translation variation and complexity accurately. In addition, long‐reads can extend the number of relevant RNAs from traditionally noncoding coding or nonfunctional species, such as lncRNAs and pseudogenes (Troskie et al., 2021; Ruiz‐Orera et al., 2014). Similarly, integration of long‐read sequencing with polysome profiling would also improve the characterization of translating mRNAs. Overall, everything points to long‐read sequencing as a necessary technology to obtain a more reliable interpretation of Ribosome profiling experiments and potentially of the assignment of MS peptides to transcripts.

Finally, it is worth highlighting that ribosome engagement and translation rates do not necessarily correlate with stable protein levels. Protein degradation, either co‐translational or posttranslational, can eliminate aberrant proteins that escaped previous quality control steps and tune cellular activity in specific conditions by modulating levels of functional proteins (Liu, Beyer, & Aebersold, 2016; Joazeiro, 2019). Thus, despite all the advances to link the transcriptome variations to protein production and function, we still need to measure the proteome. Proteomics has been limiting so far to provide evidence of these protein variants for which there are multiple other sources of evidence. On the other hand, emerging single‐molecule protein sequencing technologies (Alfaro et al., 2021) could give a genuinely unbiased measurement of proteomes and, more precisely, unveil the extent to which alternative splicing impacts the proteome and, thereby, the function of genes.

## CONFLICT OF INTEREST

The authors declare no conflicts of interest.

## AUTHOR CONTRIBUTIONS

**Marina Reixachs‐Solé:** Conceptualization (lead); data curation (lead); resources (lead); visualization (lead); writing – original draft (lead); writing – review and editing (lead). **Eduardo Eyras:** Conceptualization (supporting); writing – original draft (supporting); writing – review and editing (supporting).

## RELATED WIREs ARTICLES

The evolution of posttranscriptional regulation

Microexons: Discovery, regulation, and function

Intron retention and its impact on gene expression and protein diversity: A review and a practical guide

## Data Availability

Data sharing is not applicable to this article as no new data were created or analyzed in this study.
